# Genomic Insights into Genetic Diversity and Adaptation of Nanyang Cattle: Implications for Conservation and Breeding

**DOI:** 10.3390/ani15203033

**Published:** 2025-10-19

**Authors:** Yan Zhang, Xian Liu, Jiakun Liu, Tong Fu, Hetian Huang, Mingpeng Han, Dong Liang, Tengyun Gao

**Affiliations:** 1 College of Animal Science and Technology, Henan Agricultural University, Zhengzhou 450046, China; zhangy20210000@163.com (Y.Z.); futong2004@126.com (T.F.); xh722109@163.com (H.H.); 2 Henan Animal Husbandry Station, Zhengzhou 450008, China; liuxian641@163.com; 3 Tongbai County Agriculture and Rural Bureau, Nanyang 474750, China; liujk_ny@163.com; 4 Henan Provincial Seed Industry Development Center, Zhengzhou 450046, China; mingpeng999@163.com

**Keywords:** Nanyang cattle, whole-genome, population structure, artificial selection, adaptability preservation

## Abstract

The conservation of agricultural animal resources holds significant importance for addressing future breeding demands and promoting sustainable utilization of regional livestock genetic resources. As one of China’s five major fine cattle breeds, Nanyang cattle exhibit notable characteristics, including strong adaptability and a docile temperament. Through whole-genome comparative analysis of the Nanyang cattle core conservation population and free-ranging populations in mountainous areas, this study systematically investigated the genetic diversity, population structure, and adaptive evolutionary mechanisms of this indigenous Chinese cattle breed. Population structure analysis revealed that both groups belong to a single genetic lineage of Nanyang cattle without significant genetic differentiation; however, the free-ranging population demonstrated a slower linkage disequilibrium decay rate. Combined *F*_ST_ and θπ ratio analyses identified 170 positively selected genes, with KEGG enrichment highlighting glutamatergic synapse and African trypanosomiasis immune pathways. PLCB4, as a shared gene across both pathways, may exert pleiotropic functions in environmental adaptation. The core conservation population likely exhibits reduced diversity in neuroplasticity-related genes under artificial selection, whereas the free-ranging population retained more genes associated with environmental adaptation and immune response. These findings provide genomic evidence for formulating differentiated conservation strategies for Nanyang cattle, necessitating balanced co-optimization of production trait breeding and natural adaptability preservation.

## Simple Summary

Nanyang cattle are one of China’s most valuable local breeds, known for their strength, calm temperament, and ability to survive in harsh environments. Yet, like many traditional animals, they face threats from modern farming practices that can reduce genetic diversity, meaning the range of traits passed from one generation to the next. In this study, we compared two groups of Nanyang cattle: one maintained in an official conservation program and another living freely in mountain regions. We found that both groups share the same origins, but the free-ranging cattle preserved more natural variation and genes linked to disease resistance and adaptation, while the conservation group showed stronger effects of human selection for traits such as growth. Notably, 170 genes were identified as playing important roles in environmental adaptation. These results show that conserving Nanyang cattle requires a careful balance between improving productivity and preserving natural resilience. Protecting the genetic resources of this breed will not only help today’s farmers but also safeguard future food security and ensure the sustainability of livestock farming.

## 1. Introduction

Domestic cattle (Bos primigenius taurus) are taxonomically classified into two major lineages: taurine (Bos taurus) and zebu (Bos indicus), both belonging to the genus Bos within the family Bovidae of the order Artiodactyla [1]. As one of the most globally significant domesticated animals, the conservation of cattle genetic resources holds exceptional biological and agricultural importance [2]. With wild ancestors (e.g., the aurochs, Bos primigenius) now extinct, their genetic diversity relies entirely on extant populations, establishing domestic cattle as a model system for biodiversity conservation studies. Livestock breeds constitute intentionally developed subpopulations shaped by founder events, genetic isolation, and directed selection, thereby preserving rare genetic adaptations to specific environments. This conservation paradigm manifests most notably in autochthonous breeds such as the low-input foraging-adapted Buša cattle of the Balkans [3], the tick-resistant Criollo cattle of the Americas [4], and the high-altitude acclimatized Tibetan cattle of China [5]. Collectively, these examples highlight the pivotal role of local breeds as living repositories of genetic and adaptive biodiversity.

China harbors one of the world’s richest repositories of bovine genetic resources, characterized by millennia of domestication and extensive variation within Bos taurus populations [6,7]. Through millennia of natural and artificial selection, including targeted hybridization events between Bos taurus and Bos indicus subspecies, numerous autochthonous cattle breeds have emerged with distinct adaptations to diverse ecological zones. Among these, the Nanyang cattle—recognized as one of China’s five principal yellow cattle breeds and historically pivotal as draft animals in agrarian societies—holds inclusion in the National Catalog of Livestock and Poultry Genetic Resources (Supplementary Figure S1). Population genetic analyses demonstrate that Nanyang cattle descend from hybrid ancestry between Bos taurus and Bos indicus lineages [8], conferring resilience to environmental stress and robust production potential. To safeguard this valuable genetic resource, the local government established a conservation farm dedicated to the systematic preservation and breeding of Nanyang cattle, while simultaneously implementing policies that encourage farmers to continue raising and utilizing the breed. After several decades of development under these contrasting management and rearing regimes, the two populations may have diverged in terms of genetic diversity and adaptive potential. To verify this hypothesis, we conducted whole-genome resequencing (WGS) to systematically evaluate the genetic structure and adaptive potential of these two Nanyang cattle populations.

Recent advances in WGS technology have revolutionized livestock genomics by overcoming the limitations of traditional molecular markers. WGS enables comprehensive detection of structural variants and selection signatures across the entire genome [9,10], thereby providing powerful insights into adaptive mechanisms of indigenous breeds. As sequencing costs continue to decline alongside refinements in bioinformatic methodologies, this approach has been successfully deployed in model systems ranging from thermotolerance adaptation in Chinese cattle to high-altitude acclimatization in Tibetan cattle [11,12], demonstrating that environmental adaptation phenotypes exhibit definable genomic footprints. Such technological evolution is fundamentally reshaping research paradigms within domestic animal genetics—shifting from singular phenotypic characterization toward integrated genome-phenome analyses, and transitioning from localized marker studies to genome-wide selective sweep investigations.

Despite the long domestication history and economic importance of Nanyang cattle, little is known about how distinct management systems—government-managed conservation farms versus traditional farmer-based rearing—have shaped their genomic diversity and adaptive potential. To address this gap, we performed WGS on two representative Nanyang populations: 30 individuals from the conservation farm (nanyang_A) and 17 free-ranging individuals from mountainous regions (nanyang_B). Using the Bos taurus reference genome (ARS-UCD1.3), we characterized genome-wide variation, resolved population structure, and identified regions under selection through fixation index (*F*_ST_) and nucleotide diversity ratio (θπ ratio) analyses. Functional annotation and KEGG pathway enrichment analyses were further conducted to reveal potential adaptive mechanisms. By integrating population genomics with functional annotation, this study provides a comprehensive understanding of genetic differentiation and adaptive evolution in Nanyang cattle under contrasting management conditions. The findings offer a mechanistic foundation for evidence-based conservation strategies, adaptive trait utilization, and the sustainable genetic improvement of indigenous cattle breeds.

## 2. Materials and Methods

### 2.1. Samples

A total of 47 ear tissue samples were collected from Nanyang cattle, with all sampling procedures following strict disinfection protocols using 75% ethanol before and after tissue extraction. The cohort comprised 30 individuals from the core conservation population in Nanyang (designated nanyang_A) and 17 free-ranging individuals from smallholder herds in mountainous areas (designated nanyang_B). All samples were obtained with documented informed consent from owners, ensuring compliance with established ethical standards for subsequent genomic data analyses.

### 2.2. Sequencing

Genomic DNA was extracted from auricular tissue samples of Nanyang cattle using the phenol-chloroform method. DNA integrity and yield were assessed by agarose gel electrophoresis and quantified using a NanoDrop spectrophotometer. Qualified DNA samples were used to construct 300-bp insert size paired-end libraries, which were subsequently sequenced on the DNBSEQ-T7 platform (MGI, CN). Raw fastq files underwent quality control processing with Trimmomatic [13] to generate clean reads. These clean reads were then aligned to the Bos taurus reference genome (ARS-UCD1.3) using the BWA-MEM [14] algorithm (v0.7.17-r1188) under default parameters. PCR duplicates were marked and removed using the MarkDuplicates (v2.27.3) module in Picard (https://broadinstitute.github.io/picard/, accessed on 15 October 2025). Local realignment around indels was performed using GATK [15] v3.8′s IndelRealigner. Initial variant calling was conducted with the GATK UnifiedGenotyper module. Detected variants were filtered based on the following criteria: QD < 2.0, FS > 60.0, MQ < 40.0, MQRankSum < −12.5, ReadPosRankSum < −8.0, and SOR > 3.0. The resulting variant set was converted from VCF to PLINK format using VCFtools (v0.1.16) [16]. Finally, quality control filtering was performed using PLINK (v1.90) [17], and a total of 63,069,870 SNPs were obtained from 47 individuals. We removed SNPs with minor allele frequency < 0.05 and Hardy–Weinberg equilibrium less than 10^−6^ were excluded. Additionally, individuals with >10% missing genotypes were excluded to ensure data robustness. A total of 32,014,481 high-quality SNPs were retained for subsequent analysis.

### 2.3. Genetic Diversity Analysis

Genetic variants in the Nanyang cattle populations were functionally annotated using SnpEff 5.1d [18], with results visualized via the ggplot2 (v3.5.2) package in R [19]. Pairwise genetic distances between individuals were derived from identity-by-state (IBS) matrices generated by PLINK, followed by matrix visualization in R. For runs of homozygosity (ROH) detection, autosomal scans were performed via PLINK’s -homozyg sliding-window algorithm. Detected ROH segments were categorized into four length-based classes (300–500 kb, 500–700 kb, 700–900 kb, and >900 kb) to compute the inbreeding coefficient (F_ROH_). All resultant data were visualized using ggplot2.

### 2.4. Population Structure Analysis

A kinship matrix was constructed from genome-wide SNP data across all populations using PLINK. PCA was performed, with the first two principal components (PC1 and PC2)—capturing the highest proportion of genetic variance—serving as orthogonal axes for population stratification visualization via the ggplot2 package in R. Genetic distances between populations were computed to generate a distance matrix (tree.mat), which was subsequently converted to Newick format (.nwk) using R for phylogenetic reconstruction. The resulting topology was visualized in FigTree v1.4.4 (http://tree.bio.ed.ac.uk/software/figtree/, accessed on 8 January 2025) to illustrate evolutionary relationships. Linkage disequilibrium (LD) decay within the Nanyang cattle cohort was analyzed using PopLDdecay (v3.42) [20], where LD intensity (r^2^) between pairwise SNPs was regressed against physical distance to generate LD decay curves. Population structure inference was conducted via ADMIXTURE (v1.3.0) [21], implementing a maximum-likelihood framework to estimate ancestry proportions and identify genetic subdivisions among subpopulations.

### 2.5. Selective Signature Analysis and Functional Enrichment

Quality control of VCF-format genomic data for the Nanyang cattle cohort was performed using PLINK. Genome-wide scans for selection signatures were conducted by integrating two complementary metrics: the *F*_ST_ and θπ ratio. Both statistics were computed via sliding-window analyses (window size: 50 kb; step size: 20 kb) implemented in VCFtools. Genomic regions simultaneously ranked within the top 1% of both metrics were designated as candidate selective sweeps. Functional annotation of genes within these regions was performed using SnpEff 5.1d with a custom-built genomic annotation database. Significantly enriched KEGG pathways were identified via the KOBAS 3.0 platform [22], applying a false discovery rate (FDR) threshold of <0.05 for multiple testing correction. Resultant pathway enrichment profiles were visualized using R-based plotting packages.

## 3. Results

### 3.1. Descriptive Statistics of SNP Loci from Whole-Genome Resequencing Data

**Figure 1:**
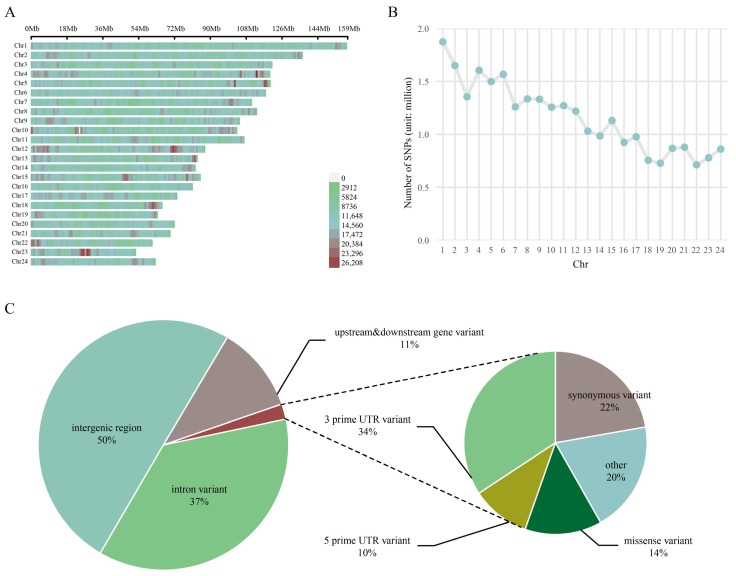
Genome-wide characterization of SNPs. (**A**) Bar plot depicting SNP density distribution across consecutive 1-Mb windows on autosomes. Color gradient reflects SNP density (SNPs/Mb), with scale bars indicating physical positions (Mb). (**B**) Histogram of SNP counts per chromosome. The dashed line denotes the genome-wide average SNP density. (**C**) Functional annotation pie chart of genomic SNPs.

Whole-genome resequencing was performed on 47 Nanyang cattle (comprising Nanyang population A: *n* = 30; population B: *n* = 17). Reads were aligned to the reference genome (ARS-UCD1.3) using BWA, achieving a mean alignment rate > 99% and mean coverage depth > 10× (Supplementary Table S1). Subsequent analyses demonstrated high accuracy and reliability based on these quality-controlled alignments. A total of 32,014,481 high-quality SNPs were identified across all samples, with chromosomal distributions illustrated in Figure 1A. SNP density exhibited homogeneity among chromosomes and showed a strong positive correlation with chromosome size: larger chromosomes contained more SNPs while smaller ones possessed fewer. Chromosome 1 harbored the maximum number of SNPs (1,873,881), as depicted in Figure 1B. Functional annotation (Figure 1C) revealed that SNPs in the Nanyang cattle genome were predominantly located in intergenic regions (50%) and intron variants (37.02%), whereas synonymous variants and missense variants accounted for lower proportions at 0.44% and 0.27%, respectively.

### 3.2. Genomic Genetic Diversity Statistics

**Figure 2:**
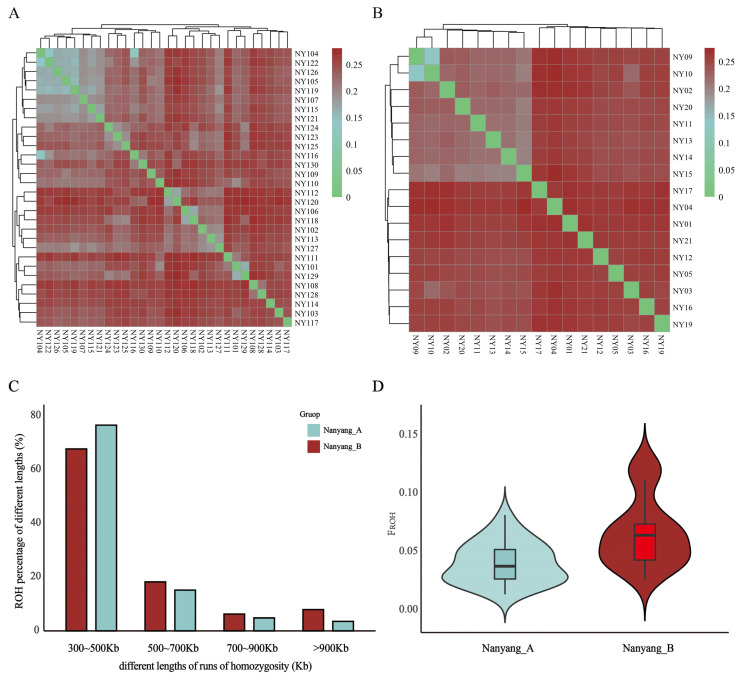
(**A**,**B**) IBS genetic distance matrices for the nanyang_A and nanyang_B. Gradient coloration in the heatmap reflects genetic divergence, with deep green regions indicating closer kinship between individuals. (**C**) Proportional distribution of ROH segments classified by length. (**D**) Boxplots of genomic F_ROH_ calculated from cumulative ROH lengths. Box boundaries denote interquartile ranges.

Analysis of IBS genetic distance matrices provides a robust approach to delineate population genetic architecture in the absence of well-documented pedigrees or ancestral genotypes. As depicted in Figure 2, pairwise IBS genetic distances within the nanyang_A ranged from 0 to 0.28, with a mean value of 0.23 ± 0.05 (Figure 2A). Similarly, distances within the nanyang_B spanned 0 to 0.28, averaging 0.23 ± 0.06 (Figure 2B). Analysis of ROH distribution revealed that nanyang_A exhibited a significantly higher proportion of short ROH segments compared to nanyang_B, whereas the latter showed a marked enrichment in long ROH segments (Figure 2C). Genomic inbreeding coefficients F_ROH_, calculated as the ratio of total ROH length to autosomal genome length (2.81 Gb, ARS-UCD1.3), demonstrated distinct patterns: nanyang_A displayed F_ROH_ values ranging from 0.014 to 0.082 (mean: 0.041 ± 0.018), while nanyang_B exhibited higher values from 0.027 to 0.125 (mean: 0.066 ± 0.030, Figure 2D). These results indicate elevated recent inbreeding in the free-ranging cohort, consistent with reduced genetic exchange in isolated mountainous habitats.

### 3.3. Population Structure Analysis

**Figure 3:**
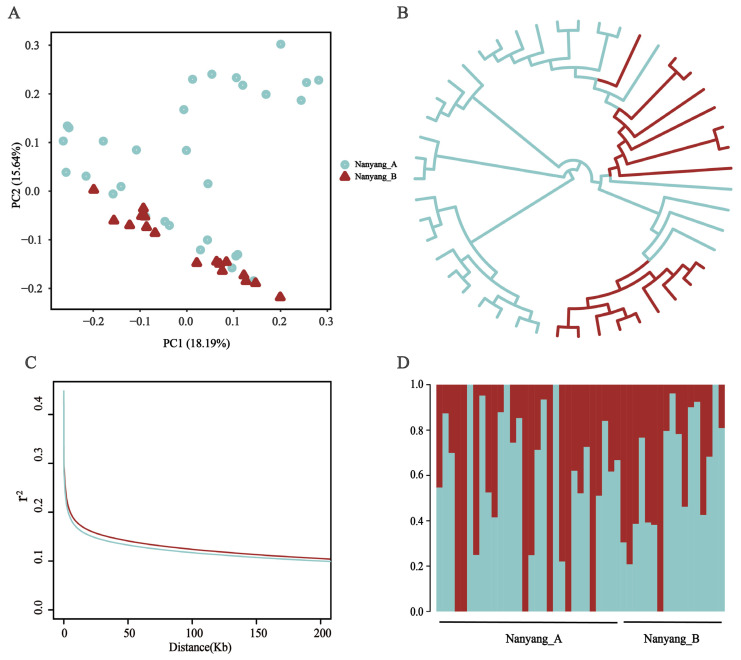
Genetic divergence analysis of Nanyang cattle subpopulations. (**A**) PCA of the nanyang_A and nanyang_B. Overlapping point clusters indicate absence of significant genetic differentiation, with the first two principal components accounting for 33.8% of cumulative variance. (**B**) NJ tree constructed from genome-wide SNP data, color-coded labels denote cohort affiliation. (**C**) LD decay profiles quantified by the decline in correlation coefficient (r^2^) between adjacent SNPs over physical distance (Mb). The slower LD decay in nanyang_B (red line) compared to nanyang_A (blue line) suggests reduced historical gene flow in the mountainous cohort. (**D**) ADMIXTURE-based population structure analysis (K = 2). Individual ancestry proportions (vertical bars) are shown along the horizontal axis.

To elucidate the genetic divergence between the nanyang_A and nanyang_B of Nanyang cattle, population genetic structure analyses were performed on 47 individuals. PCA and NJ tree construction revealed no significant genetic differentiation, with individuals from both cohorts coalescing into a monophyletic cluster (Figure 3A,B). Analysis of LD decay rates demonstrated marginally stronger SNP linkage persistence in nanyang_B relative to nanyang_A, quantified by slower r^2^ decay (Figure 3C). Ancestral component decomposition via ADMIXTURE at K = 2 further confirmed the absence of distinct genetic stratification, indicating cohesive ancestral contributions across both cohorts consistent with a unified Nanyang cattle population (Figure 3D).

### 3.4. Selective Signature Analysis and Functional Enrichment

**Figure 4:**
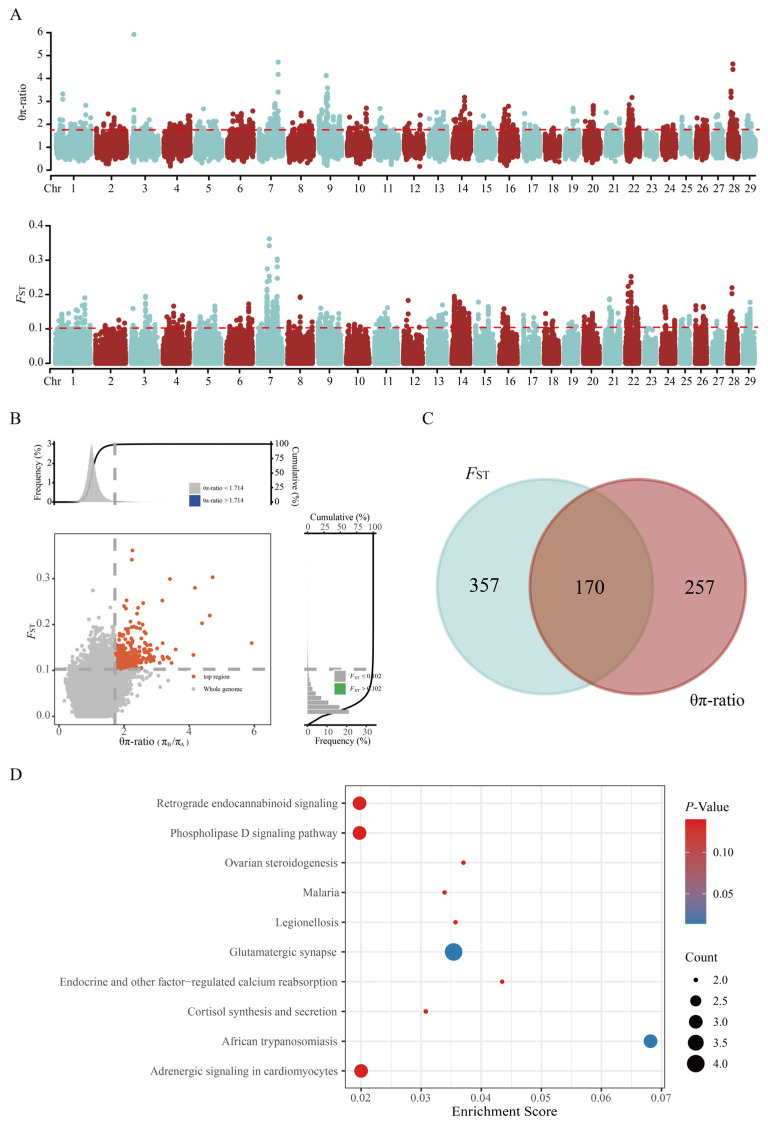
Selective signature analysis and functional enrichment of Nanyang cattle subpopulations. (**A**) Manhattan plot illustrating genome-wide selective regions between the nanyang_A and nanyang_B. (**B**) Scatter plot of *F*_ST_ versus θπ ratio. Gray dashed lines indicate the top 1% significance thresholds for each metric (*F*_ST_ = 0.102; θπ ratio = 1.714), with red circles highlighting candidate windows jointly identified by both methods. (**C**) Venn diagram of genes overlapping within the top 1% windows of *F*_ST_ and θπ ratio analyses. The intersection corresponds to the co-detected candidate genes in (**B**). (**D**) Bubble plot of KEGG pathway enrichment analysis for the 170 candidate genes. Circle area scales with gene count, and color gradient reflects enrichment significance.

To investigate genomic signatures of selection between the nanyang_A and nanyang_B of Nanyang cattle, we employed *F*_ST_ and θπ ratio analyses (Figure 4A,B). Genomic windows with signals in the top 1% of the whole-genome distribution for both metrics were defined as candidate selective sweep regions. This approach identified 527 candidate genes from *F*_ST_ peaks and 427 from θπ ratio outliers, with 170 genes overlapping between the two methods (Figure 4C). KEGG pathway enrichment analysis of these shared genes (Figure 4D, corrected *p* < 0.05) revealed significant overrepresentation in glutamatergic synapse (encompassing HOMER1, HOMER2, PLCB4, and ADCY9) and African trypanosomiasis (containing SELE, MYD88, and PLCB4). The dual involvement of PLCB4 in neuroexcitatory transmission and immune response pathways suggests its pleiotropic role in environmental adaptation mechanisms.

## 4. Discussion

Livestock genetic resources constitute a strategic foundation for ensuring national food security and revitalizing the seed industry [23]. As a nationally protected genetic resource listed in the National Catalog of Livestock and Poultry Genetic Resources, Nanyang cattle (Bos taurus) serve as a vital model for assessing conservation efficacy in indigenous Chinese breeds. In 1996, the Nanyang cattle population peaked at approximately 2.4 million head but subsequently declined. By the end of 2005, the total population had decreased to 1.918 million, including 213,000 adult bulls and 793,000 adult cows, with artificial insemination as the predominant breeding method. The Third National Livestock and Poultry Genetic Resources Survey, completed in 2023, reported that the number of Nanyang cattle in their primary distribution areas had plummeted to just over 14,000, underscoring the urgency of conservation efforts.

Currently, to protect livestock genetic resources from the influence of exotic breeds, conservation farms typically employ closed herd breeding strategies. Therefore, studying the population structure of conserved groups is crucial for their sustainable development. The IBS-based genetic distance matrix allows analysis even when the pedigree of samples or ancestral information is unclear. In this study, the pairwise IBS genetic distances within both the core conservation Nanyang cattle population (nanyang_A) and the free-ranging mountainous population (nanyang_B) ranged from 0 to 0.28, with no significant differences in mean values. Based on these results, we infer that nanyang_A and nanyang_B have not diverged into distinct populations.

ROH refers to continuous homozygous segments in diploid genomes, where long ROH segments signify recent inbreeding events, while short segments reflect ancestral genetic contributions [24,25]. The F_ROH_ demonstrated significantly lower values in nanyang_A than in nanyang_B. Within the core conservation group, deliberate measures to protect Nanyang cattle genetic resources actively prevent large-scale inbreeding; in contrast, free-ranging herds exhibit inherently elevated inbreeding levels (Figure 2D) due to farmers’ economic incentives favoring minimal financial investment for returns, resulting in natural mating practices that exacerbate genetic homogeneity.

Population structure analyses revealed that both the nanyang_A and nanyang_B of Nanyang cattle share congruent genetic backgrounds without significant divergence, confirming their classification as a unified genetic population. Although Nanyang cattle are listed in the National Catalog of Livestock and Poultry Genetic Resources with governmental policy and financial support [26], conservation programs must simultaneously preserve genetic diversity and prevent excessive inbreeding [27]. This necessitates the strategic introduction of locally sourced Nanyang cattle individuals to maintain acceptable inbreeding coefficients within managed breeding populations.

To investigate the selective signatures between the core conservation population and free-range population, we performed selection signal analyses on nanyang_A and nanyang_B using *F*_ST_ and θπ ratio approaches. Functional annotation of the significant genomic regions identified by both methods revealed 170 overlapping genes. KEGG enrichment analysis demonstrated significant enrichment (corrected *p* < 0.05) in two pathways: glutamatergic synapse and African trypanosomiasis. The glutamatergic synapse pathway, serving as the primary excitatory neurotransmission system in the central nervous system, is closely associated with neurological health and disorders [28,29]. Adaptive evolution of neurodevelopmental genes enhances organisms’ physiological adaptability to environmental stressors, thereby reducing stress-induced growth inhibition [30]. While direct evidence in Nanyang cattle is lacking, these genes may represent potential candidates for investigating neurological regulation of metabolism or stress responses, which could be indirectly related to production traits. Within this pathway, HOMER1 regulates myofiber differentiation [31], HOMER2 associates with microgravity-induced muscular atrophy [32], and ADCY9-mediated cAMP signaling maintains muscle mass and function [33]. These functional roles suggest that allelic variation in these genes may contribute to maintaining performance under challenging environmental or management conditions. African trypanosomiasis pathway genes predominantly govern humoral immune responses: SELE acts as a lymphangiogenic receptor on lymphatic endothelial cells [34], MYD88 is essential for innate immunity, where aberrant activation promotes inflammatory cytokine production and tumor progression [35], PLCB4—a shared gene across both pathways—serves as a key regulator of IP3 and DAG, potent second messengers critical for cell proliferation and immune signaling [36,37]. We further speculate that polymorphisms in these immune-related genes may confer differential resistance to parasitic or environmental stressors in Nanyang cattle, supporting survival in free-ranging or resource-limited settings. Collectively, these analyses suggest that the identified pathways could underlie adaptive traits relevant to both metabolic efficiency and environmental resilience. While these interpretations remain hypothetical, they provide a framework for future functional studies, such as stress-challenged transcriptomics or metabolomics, to validate how these variants influence physiological and adaptive responses.

This genomic investigation delineates the genetic characteristics of Nanyang cattle core conservation (nanyang_A; *n* = 30) and free-ranging mountainous populations (nanyang_B; *n* = 17), yet methodological limitations warrant refinement. Constrained by cohort size and sampling conditions, the restricted sample size, coupled with limited environmental heterogeneity and potential gene flow between cohorts, may compromise the statistical power of population genetic inferences [38,39]. This limitation reduces the ability to detect selection signals and population differentiation, particularly for variants with small effects or uneven distribution, which may result in genuine signals being overlooked. In addition, it can amplify the impact of genetic drift and sampling error, broadening confidence intervals for allele frequency estimates and increasing the likelihood of false positives. Furthermore, we acknowledge that aligning reads to the Bos taurus ARS-UCD1.3 reference genome may introduce slight reference allele bias due to the Bos indicus ancestry of Nanyang cattle. While genome-wide patterns such as *F*_ST_ and θπ ratio are expected to remain largely robust, local diversity estimates and selection signals could be subtly affected. Future studies could consider using a B. indicus reference genome or a pangenome approach to further validate these findings. Subsequent studies should quantify genetic relatedness between populations, expand sampling scales to enhance the reliability of selection signal detection, and incorporate phylogenetically proximal local breeds to elucidate gene flow dynamics. Although neuro-immunological candidate genes were identified, their mechanistic roles in shaping environmental adaptations remain unverified experimentally. Future investigations could incorporate specific multi-omics approaches, such as transcriptomics under stress-challenged conditions to reveal dynamic expression patterns of key genes, proteomics to resolve regulatory networks of immune- and neuro-related pathways, and metabolomics to trace metabolite changes associated with environmental adaptation. At the functional level, candidate genes may be validated through gene editing techniques (e.g., CRISPR/Cas9), cell-based assays, and animal models, thereby providing stronger evidence for their causal roles in shaping adaptive traits and informing genetic improvement. Such integrative strategies will facilitate the comprehensive exploitation of Nanyang cattle genetic resources and support the sustainable utilization of this regionally distinctive livestock breed.

As a valuable indigenous genetic resource in China, Nanyang cattle exhibit distinctive advantages in adaptability and genetic diversity [26,40]. Against the backdrop of progressive depletion in existing bovine genetic resources, research on this precious local breed enables a comprehensive investigation of its genetic architecture and adaptability mechanisms, providing a solid foundation for future studies targeting economically important traits. Looking ahead, conservation of Nanyang cattle should integrate genomic insights to implement differentiated strategies: introducing gene flow from free-ranging mountainous individuals into the core conservation population to enhance genetic diversity, while establishing ecological reserves to preserve the natural adaptive traits of the free-ranging cohort. In parallel, developing a genomic resource bank to regularly monitor key adaptive genes (e.g., PLCB4), balancing economic traits with stress resistance in breeding programs, and promoting long-term preservation through policy support and farmer participation will ensure sustainable utilization of this valuable genetic resource.

## 5. Conclusions

This study employed whole-genome analysis to uncover genetic distinctions within Nanyang cattle populations under different environments. Both nucleotide diversity and inbreeding coefficients in the core conservation population were lower than those in the free-ranging population. Population structure analysis confirmed that both groups belong to the same genetic lineage. Selection signature analysis identified 170 positively selected genes significantly enriched in glutamatergic synapse and immune-related pathways, with the PLCB4 gene potentially exerting pleiotropic functions in environmental adaptation. Interpretations are constrained by the relatively small sample size, which may limit generalizability, and the functional links of candidate genes remain speculative, warranting validation through future functional assays. Despite these limitations, our findings provide a valuable genomic foundation for guiding conservation strategies. Future initiatives could integrate key candidate gene information with enhanced breeding and mating management to balance production trait improvement with the preservation of natural adaptability, thereby promoting sustainable population development.

## Data Availability

The data presented in this study are available upon request from the corresponding author.

## Supplementary Materials

The following supporting information can be downloaded at: https://www.mdpi.com/article/10.3390/ani15203033/s1, Figure S1: Nanyang cattle from two populations: the core conservation population (nanyang_A, left) and the free-ranging population (nanyang_B, right); Table S1:Overview of whole genome sequencing data of Nanyang cattle.

## Abbreviations

The following abbreviations are used in this manuscript:

WGS	Whole-genome sequencing
PCA	principal component analysis
NJ	Neighbor-joining
F_ST_	Ixation index
θπ ratio	Nucleotide diversity ratio
IBS	Identity-by-state
ROH	Runs of homozygosity
F_ROH_	Inbreeding coefficient
LD	Linkage disequilibrium
