# Supplementary material for: Genomic Insights into Genetic Diversity and Adaptation of Nanyang Cattle: Implications for Conservation and Breeding
Source: Animals (Basel). 2025 Oct 19;15(20):3033. doi: 10.3390/ani15203033 (PMC12562270; doi:10.3390/ani15203033)
Supplement: Supplementary file 1 [file animals-15-03033-s001.zip › Supplementary Figure.pdf]

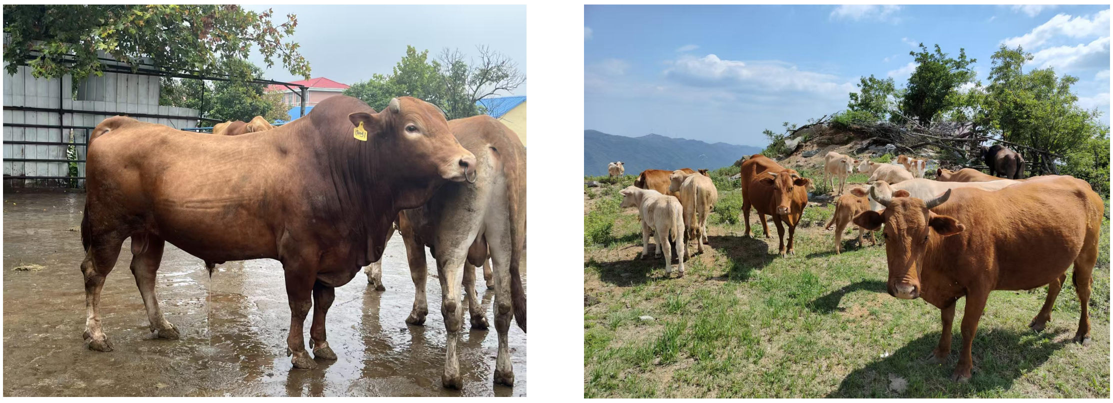

Supplementary Figure S1. Nanyang cattle from two populations: the core conservation population (nanyang_A, left) and the free-ranging population (nanyang_B, right).
